# Supplementary material for: Synthesis of Novel Sulfamethaoxazole 4-Thiazolidinone Hybrids and Their Biological Evaluation
Source: Molecules. 2020 Aug 6;25(16):3570. doi: 10.3390/molecules25163570 (PMC7464187; doi:10.3390/molecules25163570)

Synthesis of novel sulfamethaoxazole 4-thiazolidinone hybrids and their biological evaluation

Mashooq A. Bhat[a,*], Mohamed A. Al-Omar[a], Ahmed M. Naglah[b,c], Azmat Ali Khan[a]

[a]*Department of Pharmaceutical Chemistry, College of Pharmacy, King Saud University, Riyadh 11451, Saudi Arabia*

[b]*Department of Pharmaceutical Chemistry, Drug Exploration and Development Chair (DEDC), College of Pharmacy, King Saud University, Riyadh 11451, Saudi Arabia*

[c]*Peptide Chemistry Department, Chemical Industries Research Division, National Research, Centre, 12622 - Dokki, Cairo, Egypt*

*Corresponding author: E-mail: mabhat@ksu.edu.sa; Phone: +966558164097

Experimental section

Chemistry

All chemicals and solvents were purchased with high purities and used without further purification. The progress of the reaction was monitored by thin layer chromatography (using silica gel 60 F-254 plates). The products were visualized with a 254 nm UV lamp. Melting points were determined by open capillary methods and are uncorrected. Products were purified by column chromatography on 100-200 mesh silica gel. The ^{1}H NMR spectra were recorded on 400 spectrometers using tetramethylsilane (TMS) as an internal standard. The ^{13}C NMR spectra were recorded at 100 MHz and chemical shifts were reported in parts per million (d) relative to tetramethylsilane (TMS) as an internal standard. Coupling constant (J) values were reported in hertz (Hz). The splitting patterns of the proton are described as s (singlet), d (doublet), dd (doublet of doublet), t (triplet), and m (multiplet) in ^{1}H NMR spectroscopic analysis. The products were confirmed by ^{1}H and ^{13}C NMR spectroscopy analysis. High-resolution mass spectra (HRMS) were obtained using Agilent 6520 (QTOF) ESI-HRMS model.

Experimental protocol for biological activity

Pharmacology

Antitubercular assay

All the synthetic compounds were screened for their *in vitro* activity against MTB (ATCC 25177) and *M. bovis* BCG (ATCC 35743) using two fold dilution technique, in order to determine the actual minimum inhibitory concentration (MIC_{90}). Activity against MTB was determined through the XTT reduction menadione assay (XRMA) reading absorbance at 470 nm. The nitrate reductase (NR) assay was performed to estimate inhibition of *M. bovis* BCG by compounds.[2] Absorbance for the NR assay was measured at 540 nm. *In vitro* activity against MTB and *M. bovis* BCG at active (8 days) and dormant (12 days) stages was performed using the XRMA and NR assay, respectively, as described above. Percentage inhibition was calculated using the following formula:

% inhibition = [(control–CMP) / (control–blank)] x 100

Where 'control' is the activity of mycobacteria without compounds, 'CMP' is the activity of mycobacteria in the presence of compounds and 'blank' is the activity of the culture medium without mycobacteria.

Cytotoxicity assay

2-chloro quinoline incorporated xanthene derivatives **7a-l** were assayed for their cytotoxic effects in four different cell lines, MCF-7, HCT-116 and A549 using MTT assay[3]**.** The cell lines were maintained under standard cell culture conditions under 5% CO_2 at 37 ℃ in 95% air humidified environment. Each concentration was tested in duplicates in a single experiment. GI_{50}/GI_{90} values were calculated using OriginPro Software.

Selectivity Index:

The selectivity index was calculated by dividing the 50% growth inhibition concentration (GI_{50}) for cell lines (MCF-7, HCT 116 and A549) by the MIC_{90} for *in vitro* activity against dormant MTB and BCG.[4]

Anti-Bacterial activity

All bacterial cultures were first grown in LB media at 37 ^{0}C at 180 RPM. Once the culture reaches 1 O.D, it is used for anti-bacterial assay. Bacterial strains *E. coli* (NCIM 2688- ATCC 25292), *P. flurescense* (NCIM 2036-ATCC 13525) as gram-negative and B. subtillis (NCIM 2079-ATCC 23857), *S. aureus* (NCIM 2010- ATCC 29213) as gram-positive are used. The assay was performed in 96 well plates after 8 h. and 12 h for Gram negative and Gram positive bacteria, respectively. 0.1 % of 1 OD culture at 620 nm was used for screening,[5] 0.1 % inoculated culture was added in to each well of 96 well plate containing the compounds to be tested.

References:

1. Weng, J.; Wang, C.; Li, H.; Wang, Y. *Green Chem*. **2006**, *8*, 96
2. (a) Pardini, I.E.; Gagliardi, M.; Colone, M.C.; Stringaro, M.; Teloni, A.R.; Brunori, R.; Nisini, L.; Fattorini, R.; Giannoni, L.F. *Microbes Infect*. **2012**, *14,* 959; (b) Sarkar, S.; Sarkar, D. *J. Biomol. Screen.* **2012**, *17*, 966.
3. Ciapetti, G.; Cenni, E.; Pratelli, L.; Pizzoferrato, A. *Biomaterials* **1993**, *14*, 359.
4. Kuete, V. *Planta Med.* **2010**, *76*, 1479.
5. Singh, R.; Nawale, L. U.; Arkile, M.; Shedbalkar, U. U.; Wadhwani, S. A.; Sarkar, D.; Chopade, B. A. *Int. J. Antimicrob. Agents* **2015**, *46*, 183.

7a. ^{1}H NMR, 400 MHz, DMSO-d_6

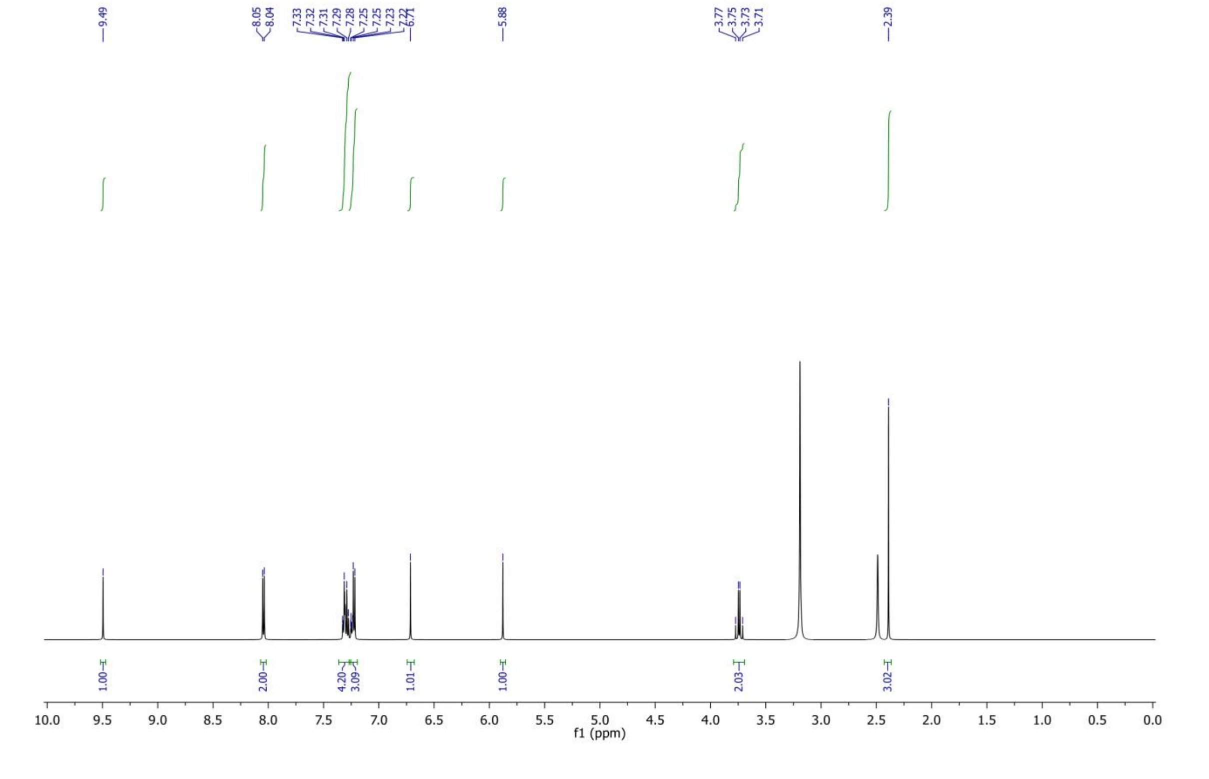

7a. ^{13}C NMR, 100 MHz, DMSO-d_6

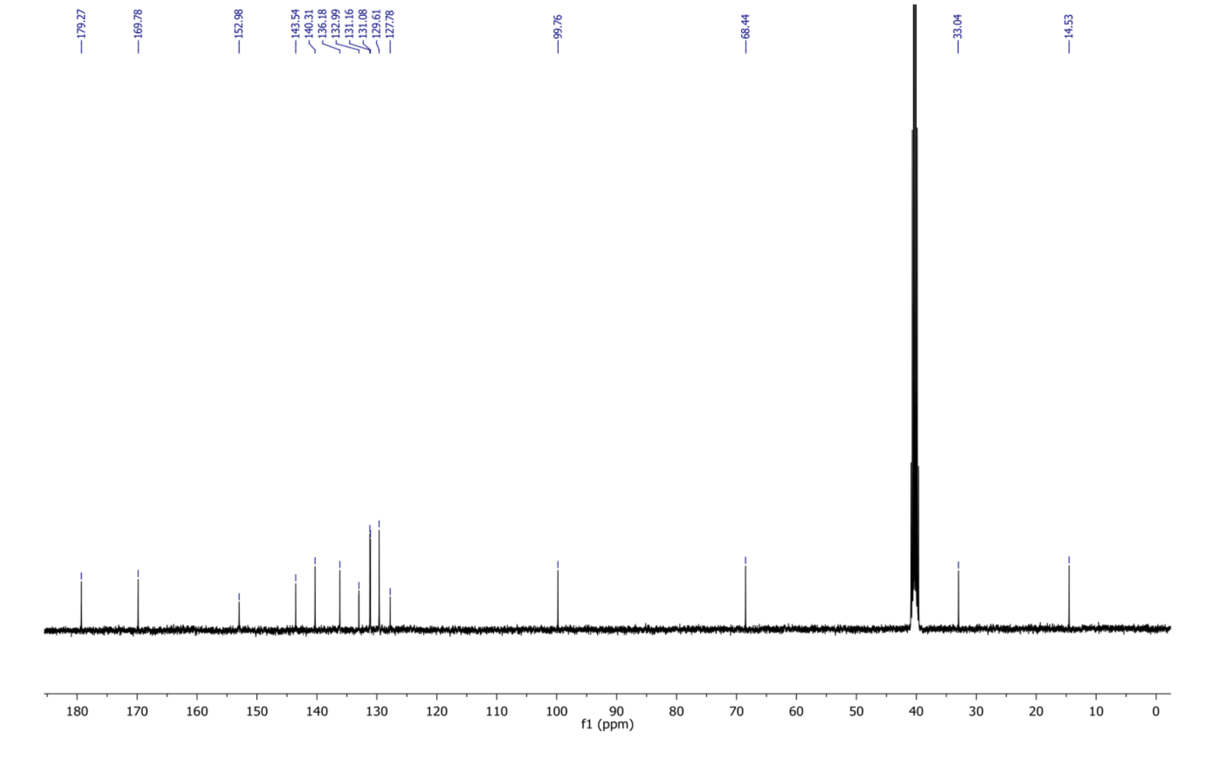

7a. HRMS

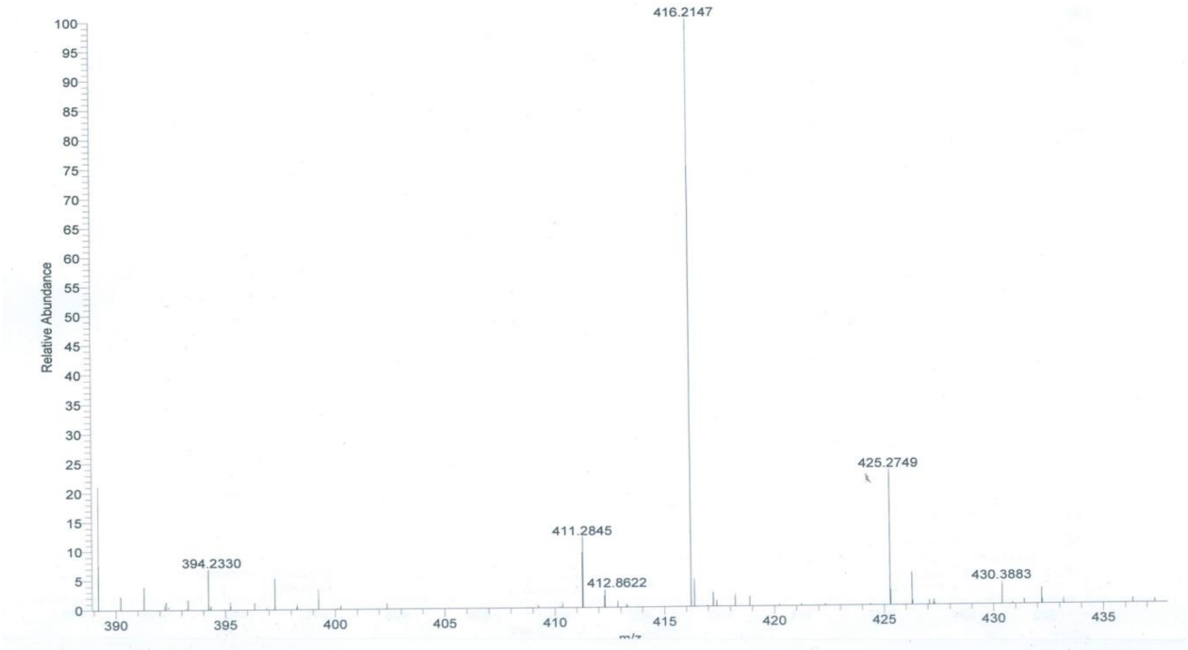

7b. ^{1}H NMR, 400 MHz, DMSO-d_6

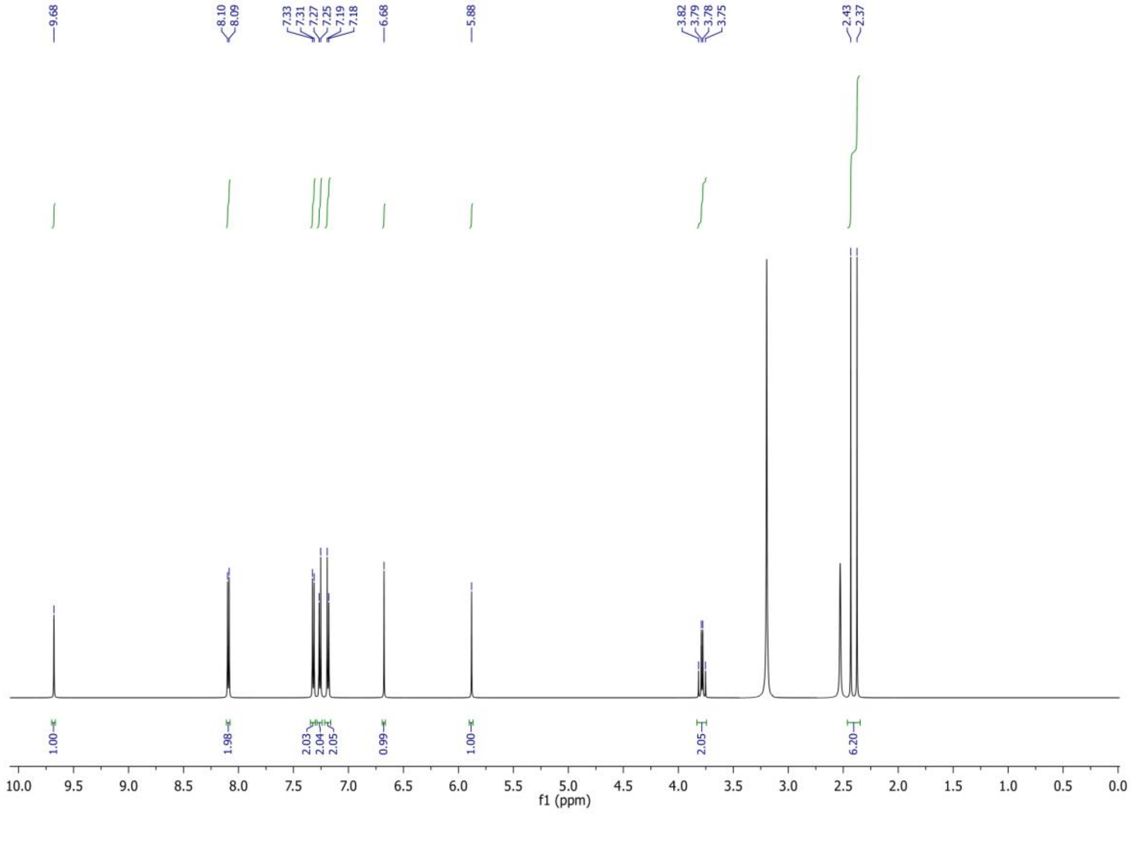

3b. ^{13}C NMR, 100 MHz, DMSO-d_6

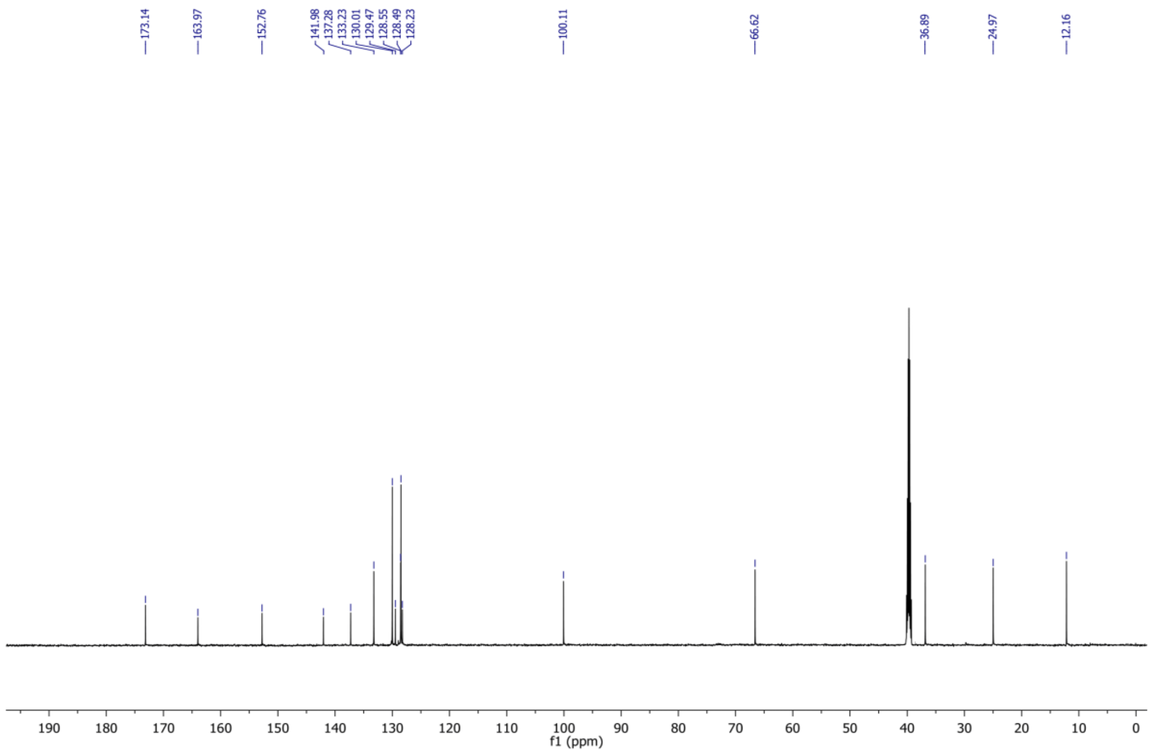

3b. HRMS

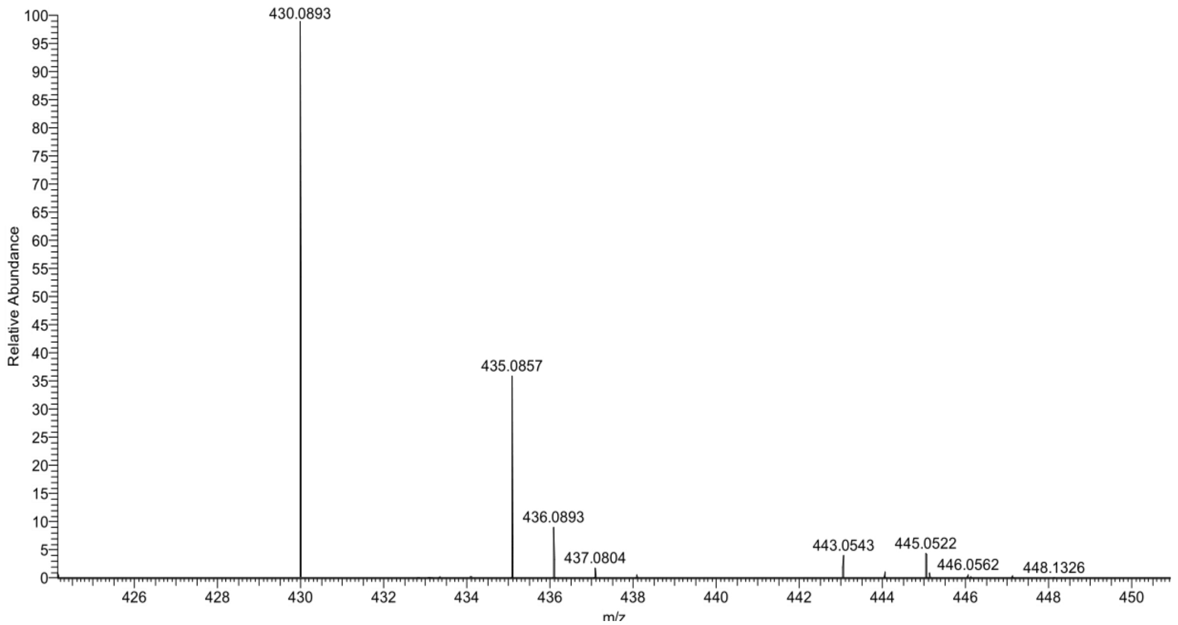

7c. ^{1}H NMR, 400 MHz, DMSO-d_6

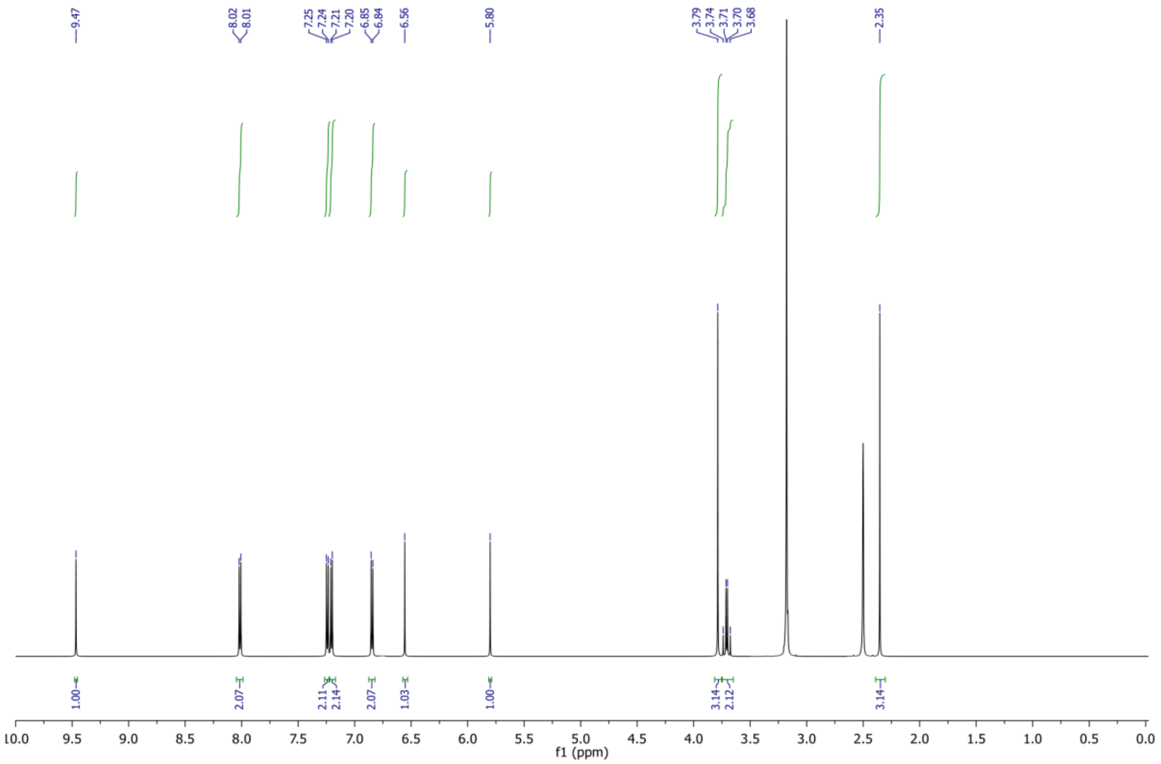

7c. ^{13}C NMR, 100 MHz, DMSO-d_6

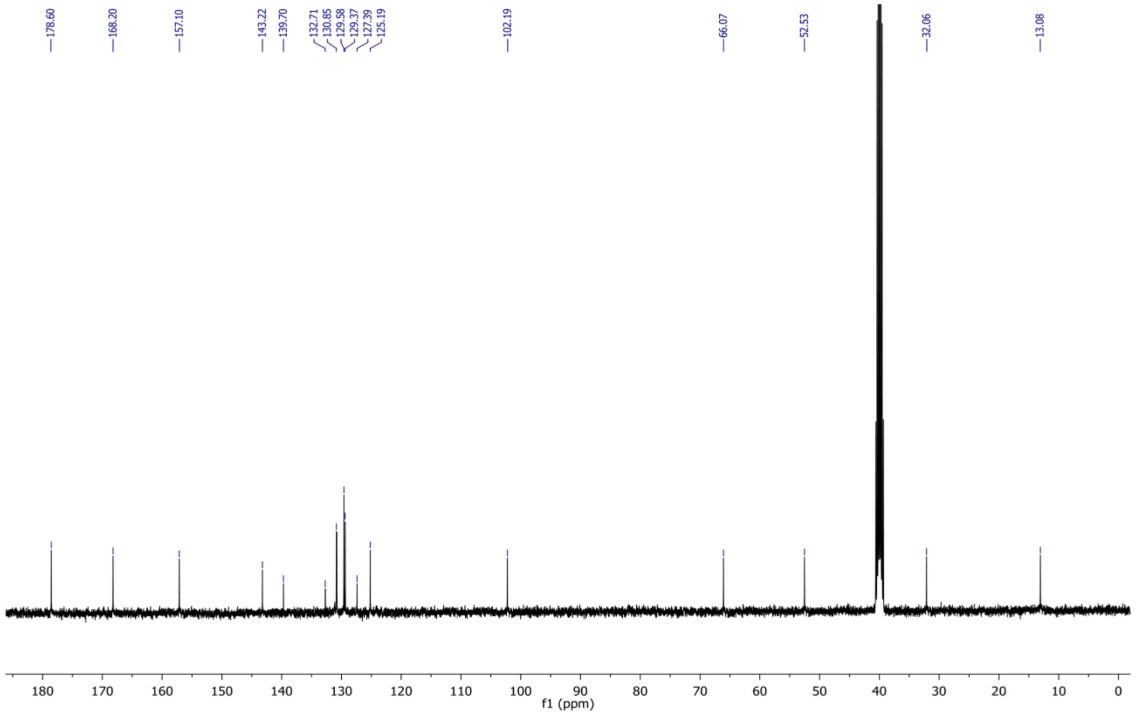

7c. HRMS

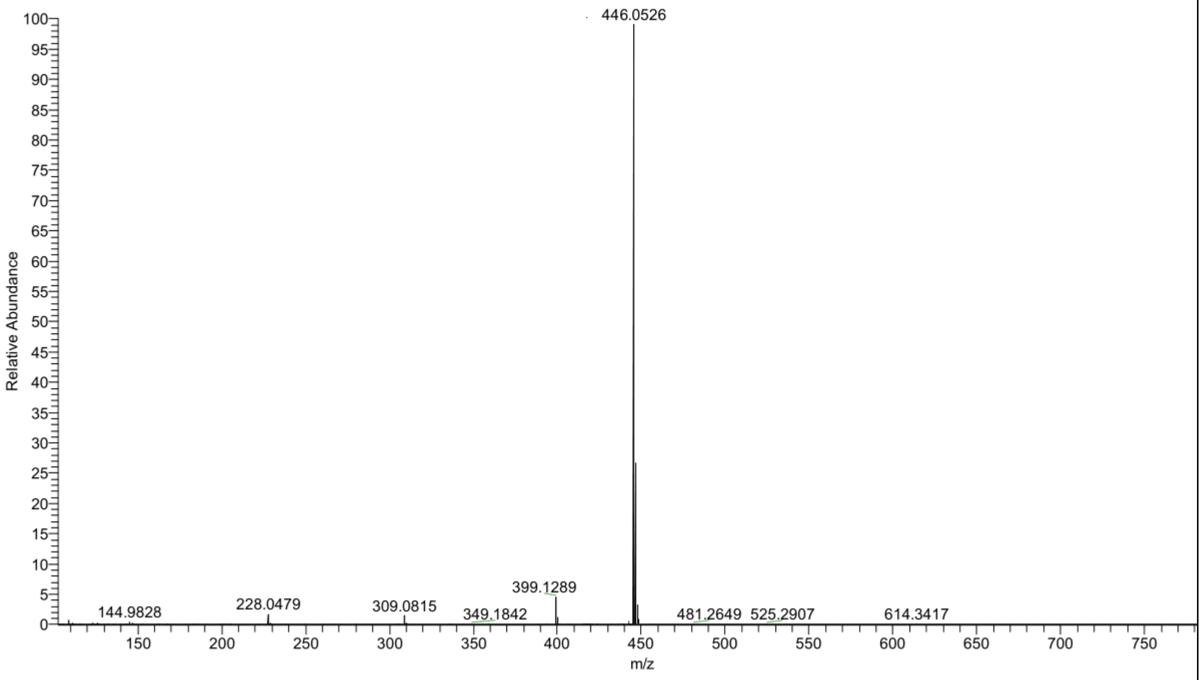

7d. ^{1}H NMR, 400 MHz, DMSO-d_6

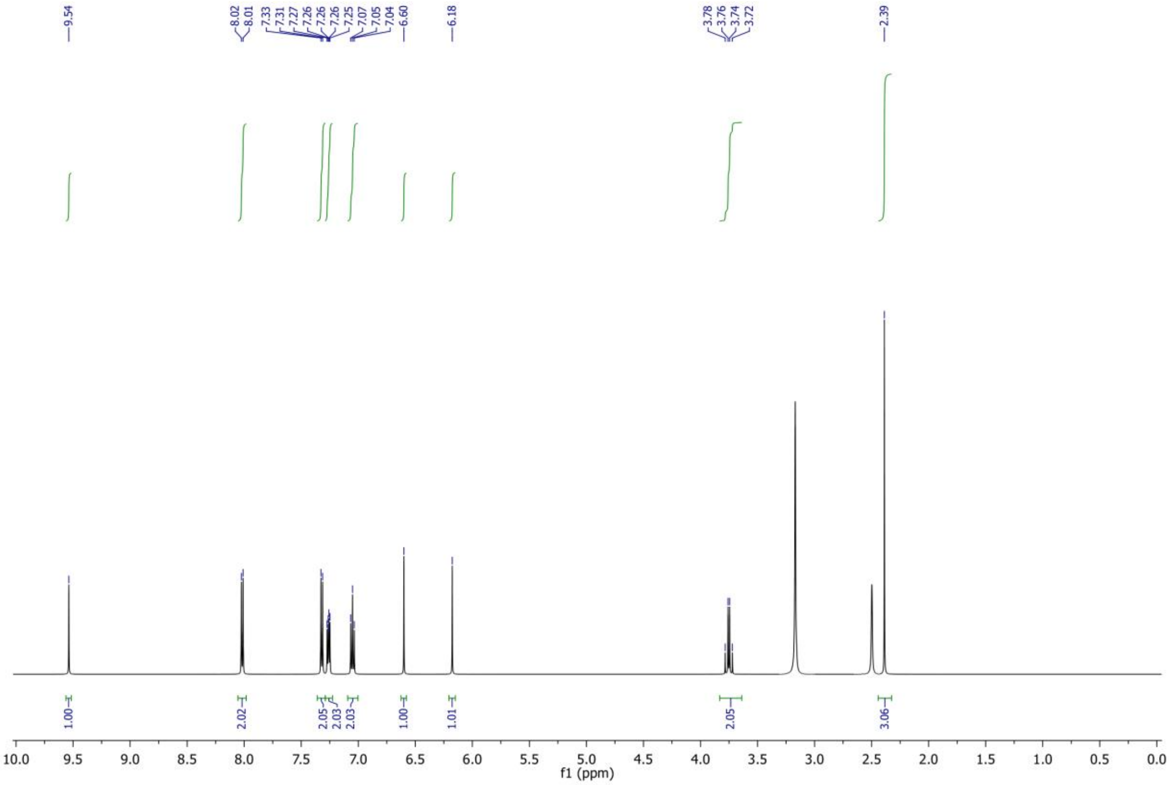

7d. ^{13}C NMR, 100 MHz, DMSO-d_6

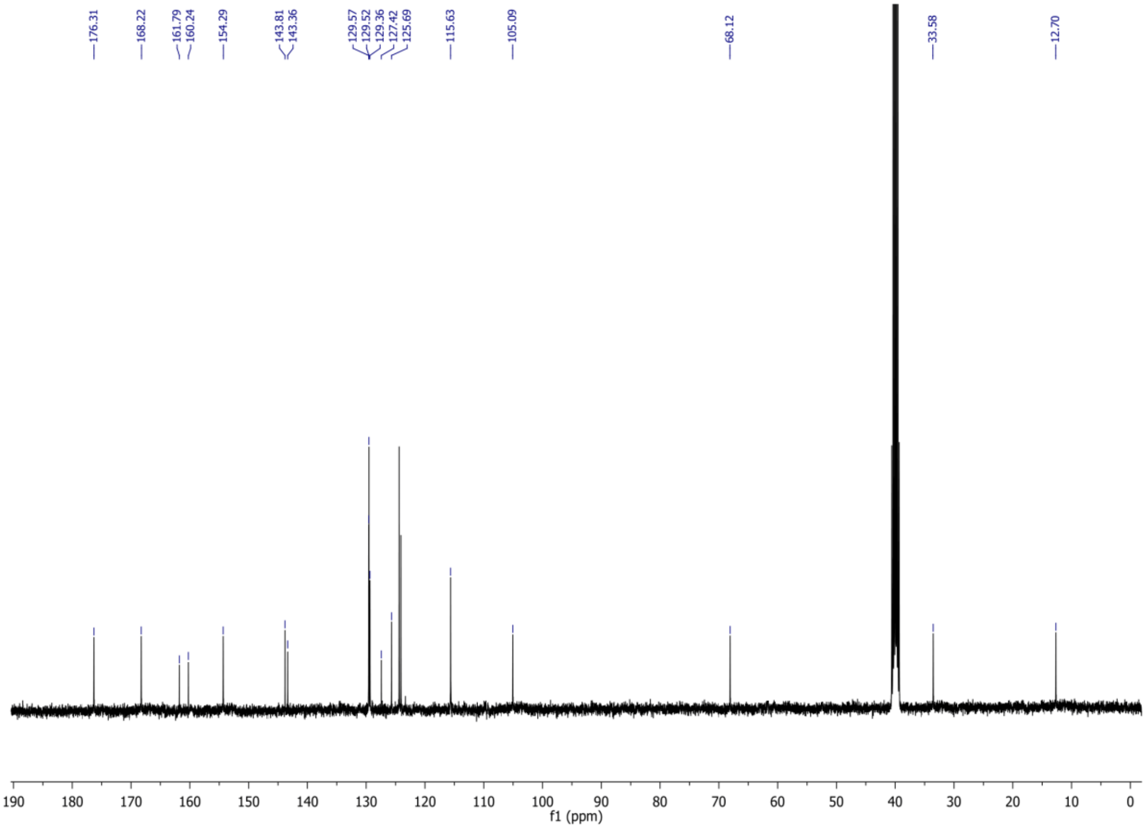

7d. HRMS

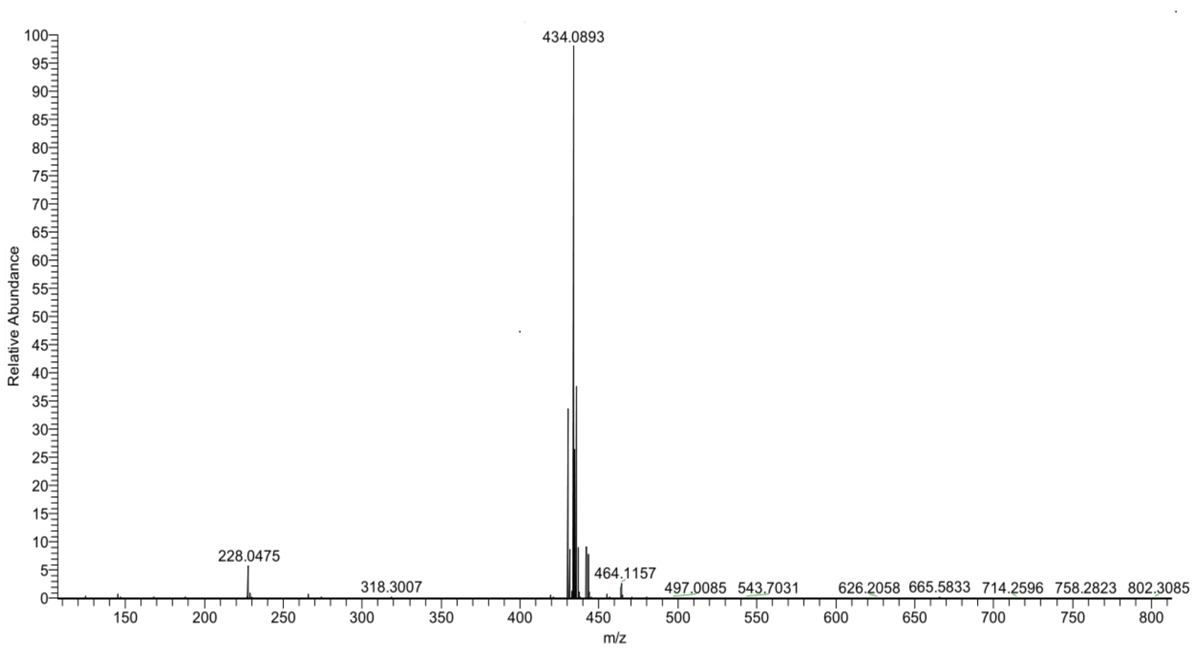

7e. ^{1}H NMR, 400 MHz, DMSO-d_6

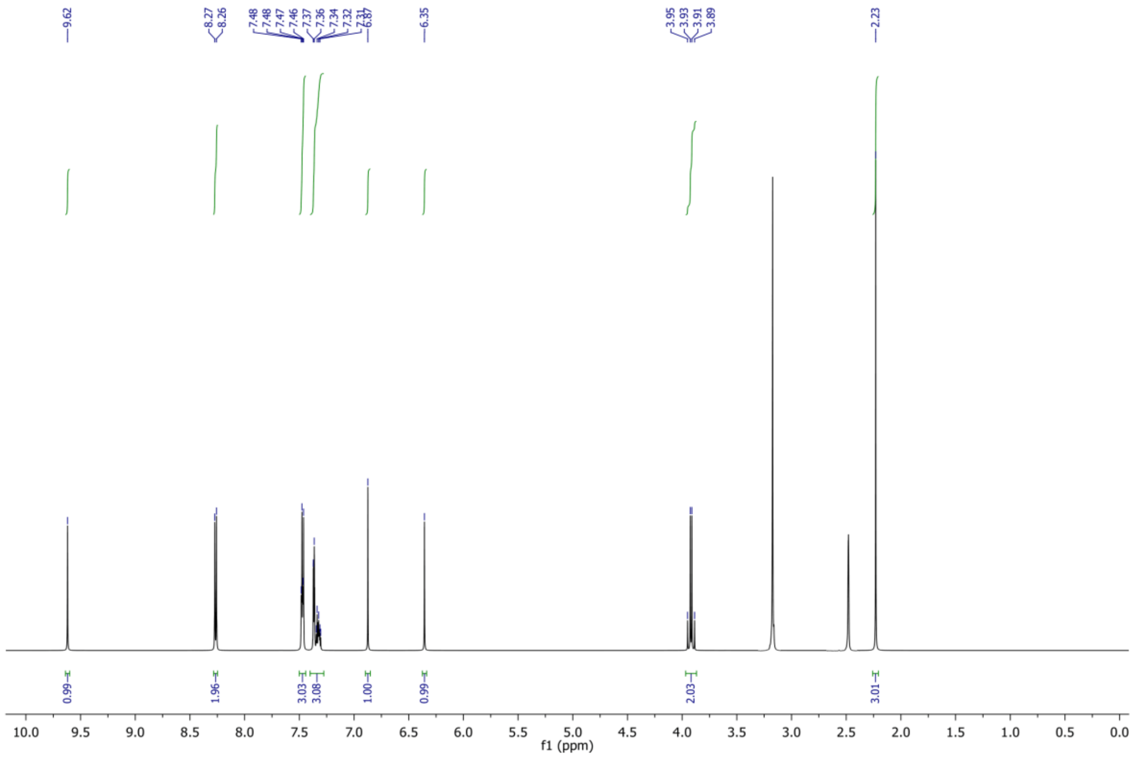

7e. ^{13}C NMR, 100 MHz, DMSO-d_6

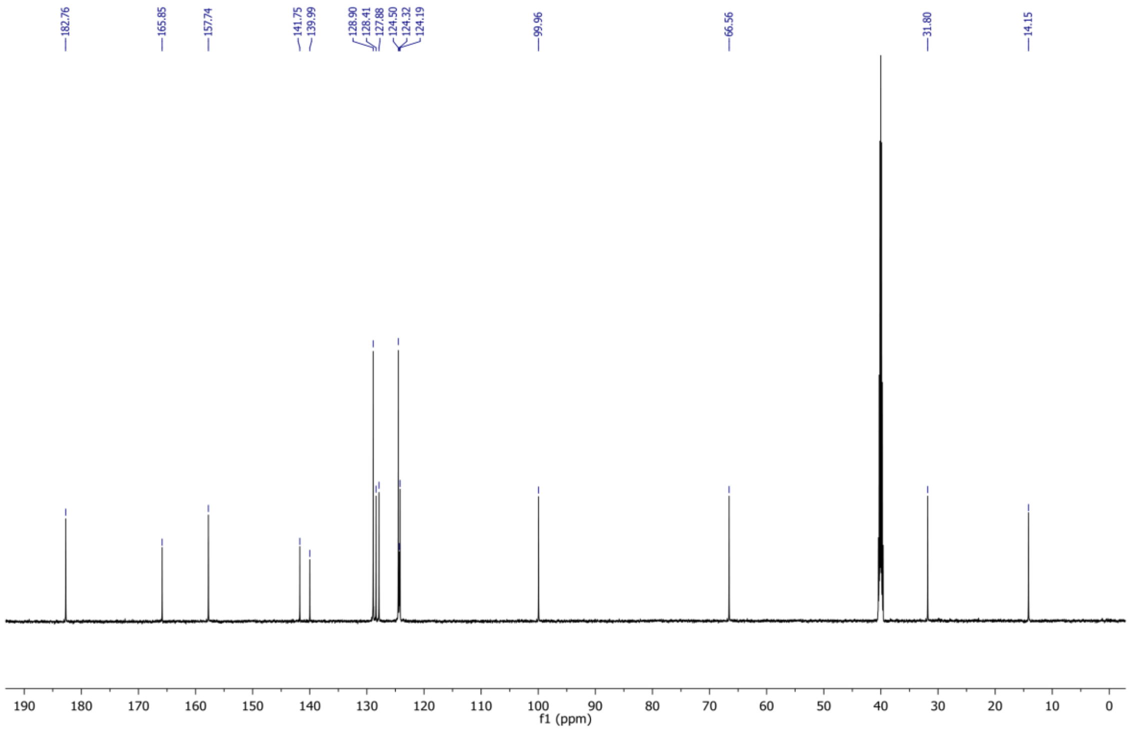

7e. HRMS

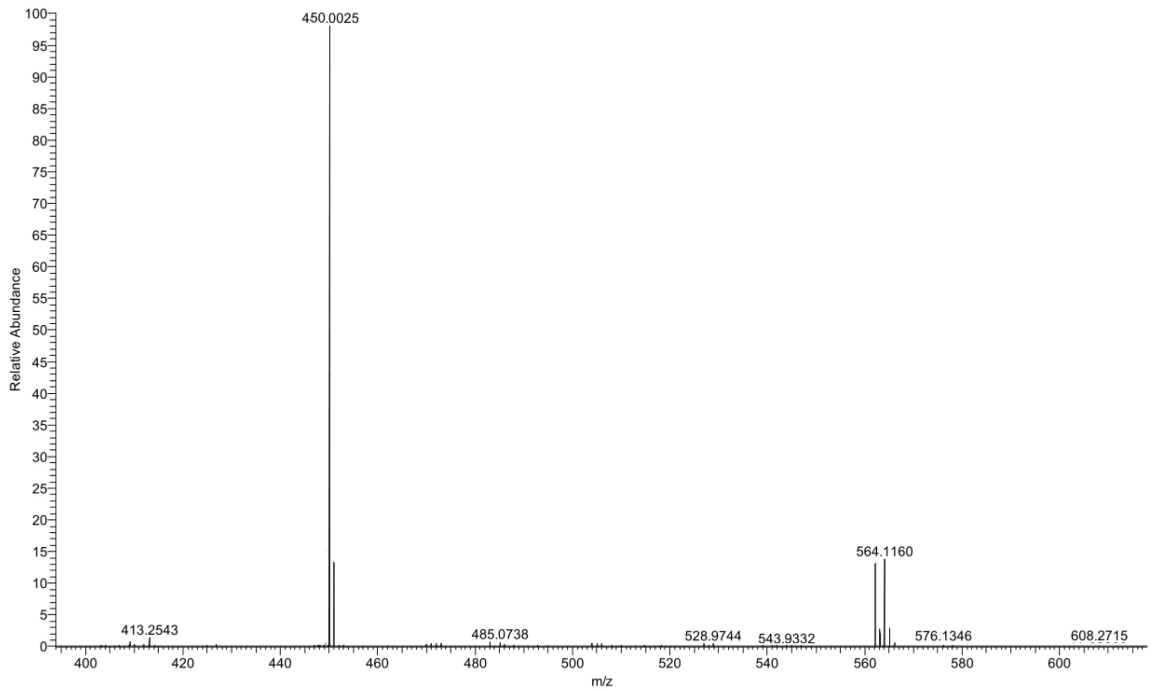

7f. ^{1}H NMR, 400 MHz, DMSO-d_6

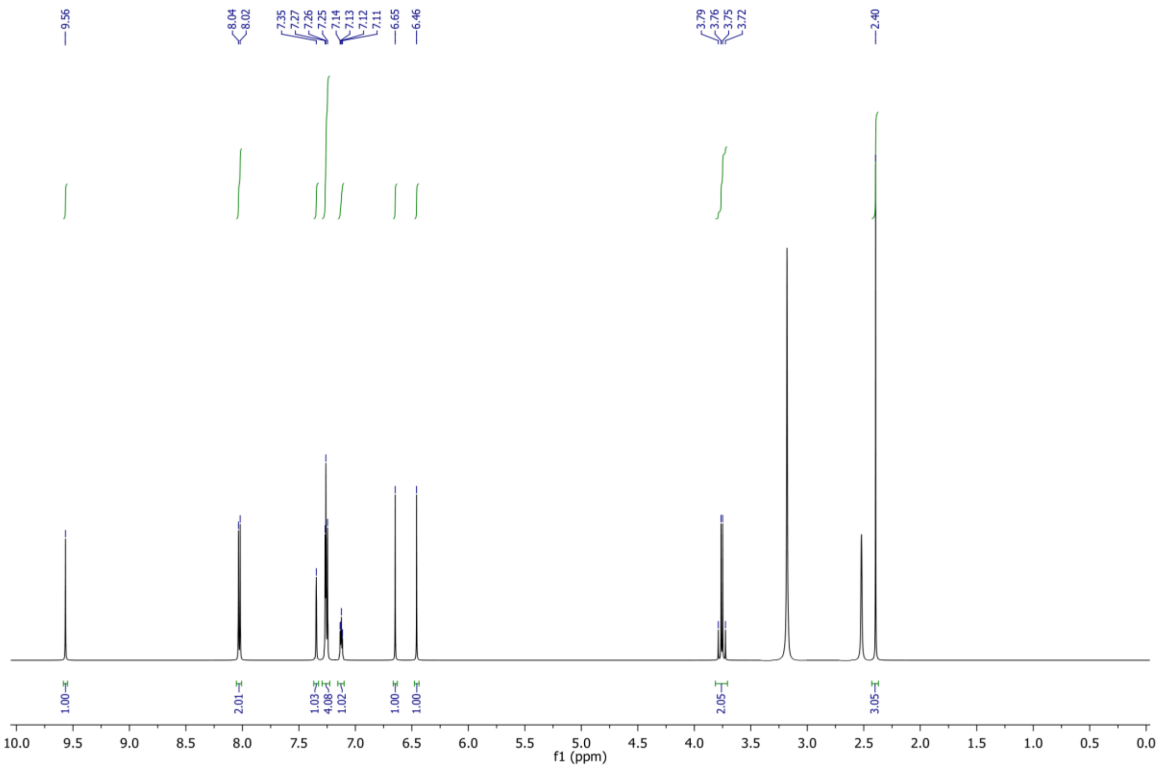

7f. ^{13}C NMR, 100 MHz, DMSO-d_6

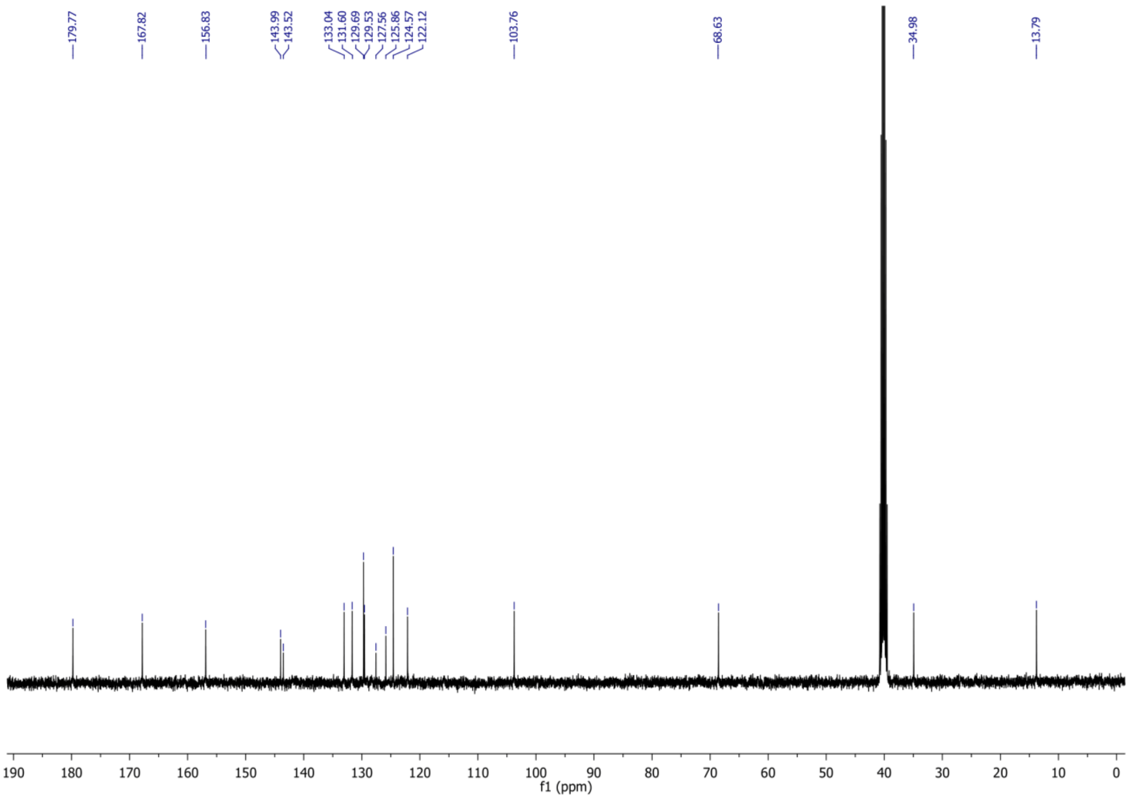

7f. HRMS

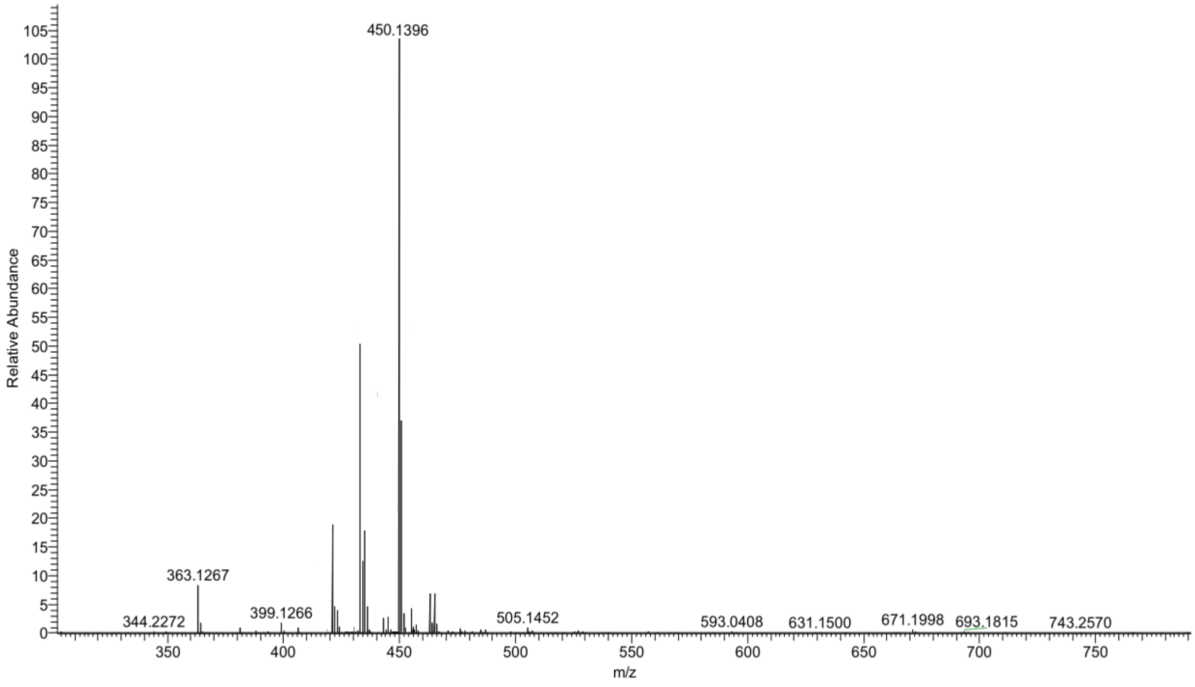

7g. ^{1}H NMR, 400 MHz, DMSO-d_6

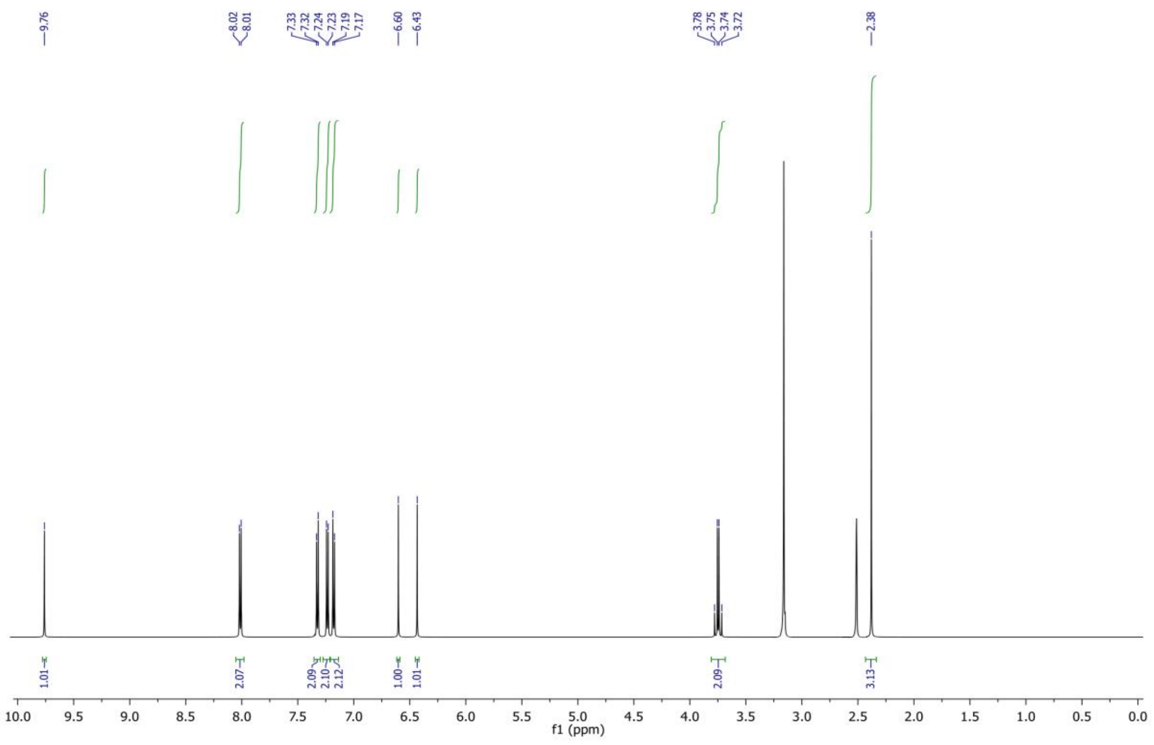

7g. ^{13}C NMR, 100 MHz, DMSO-d_6

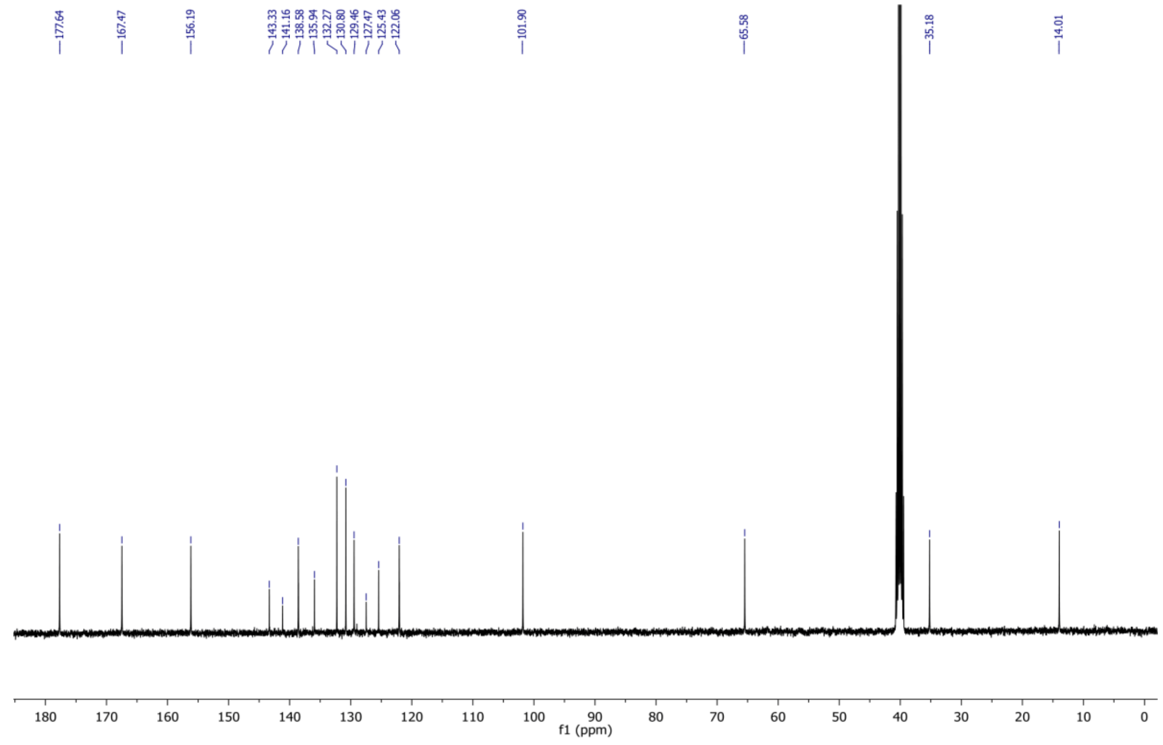

7g. HRMS

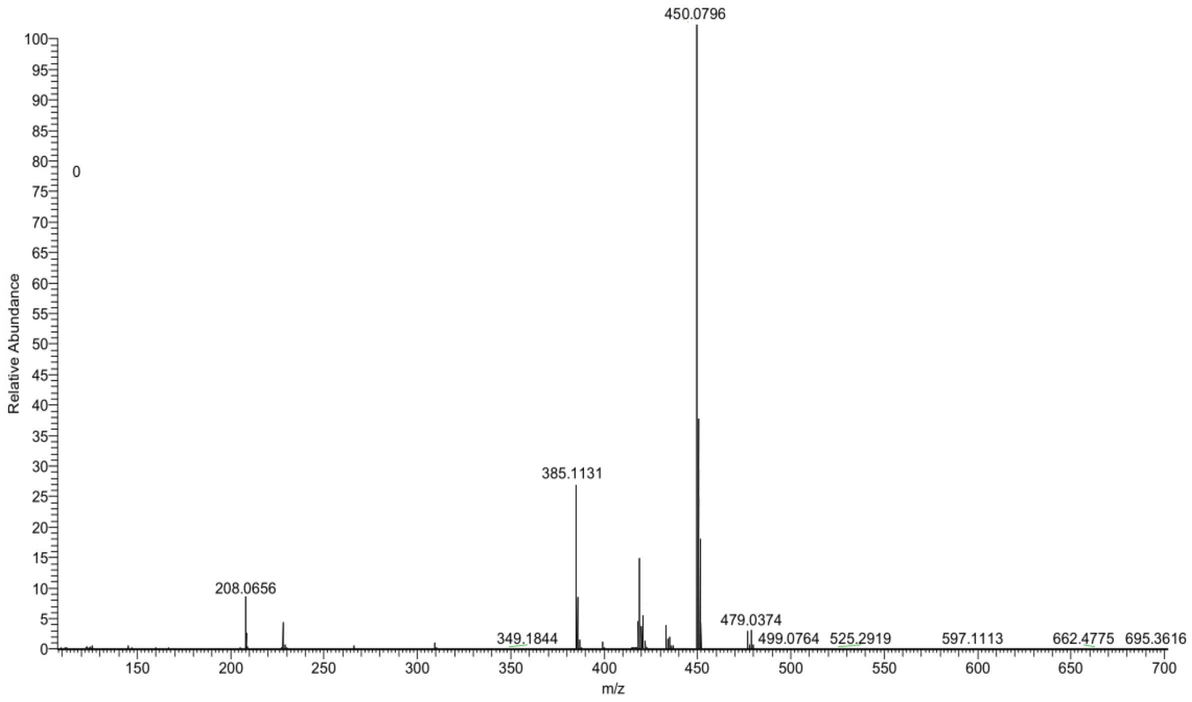

7h. ^{1}H NMR, 400 MHz, DMSO-d_6

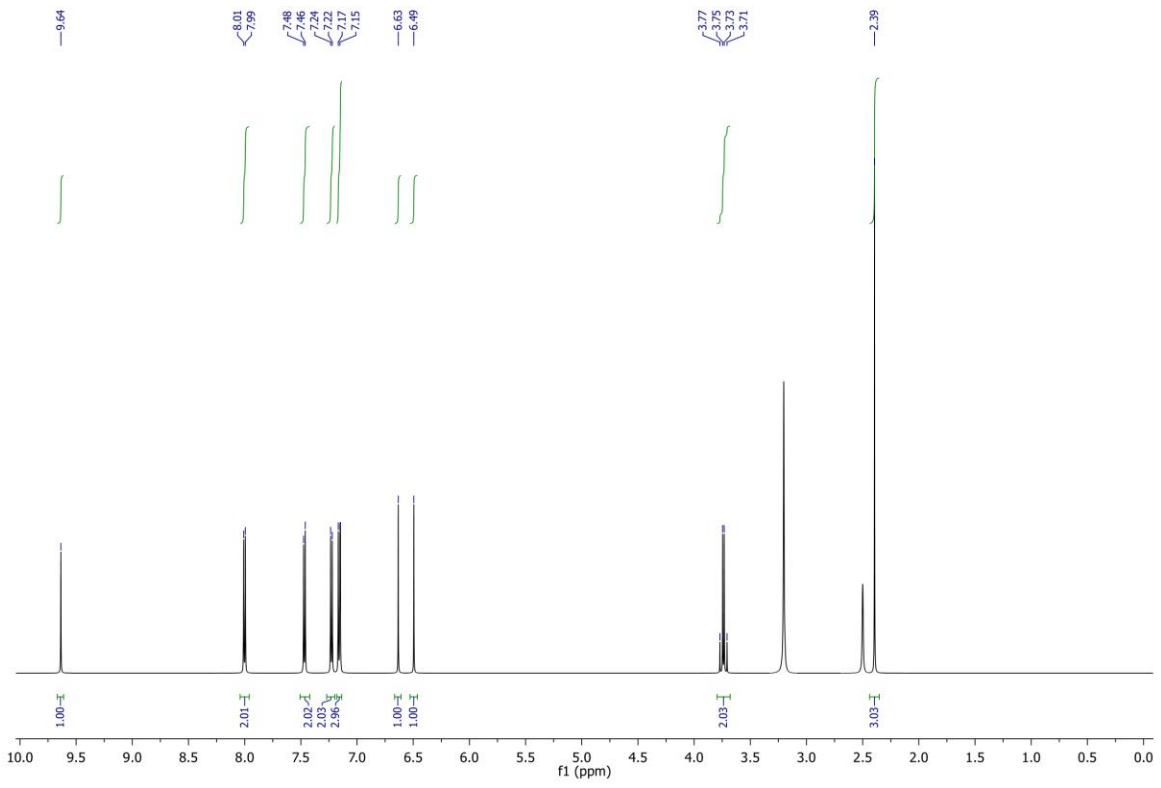

7h. ^{13}C NMR, 100 MHz, DMSO-d_6

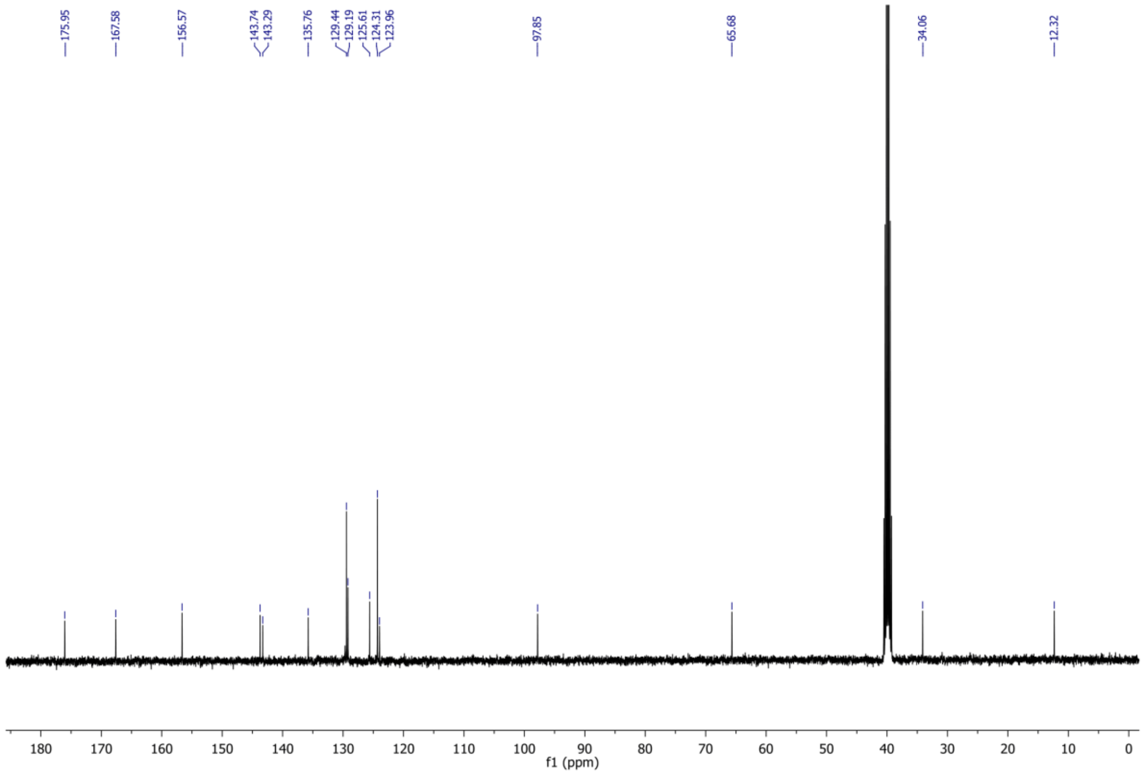

7h. HRMS

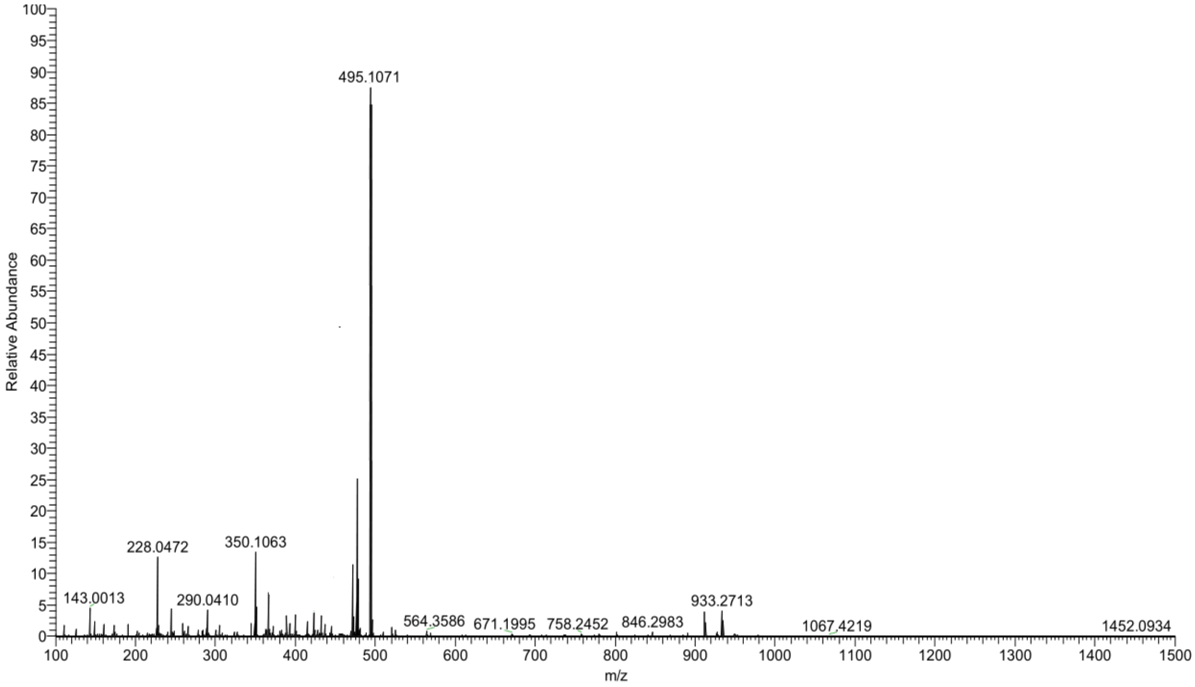

7i. ^{1}H NMR, 400 MHz, DMSO-d_6

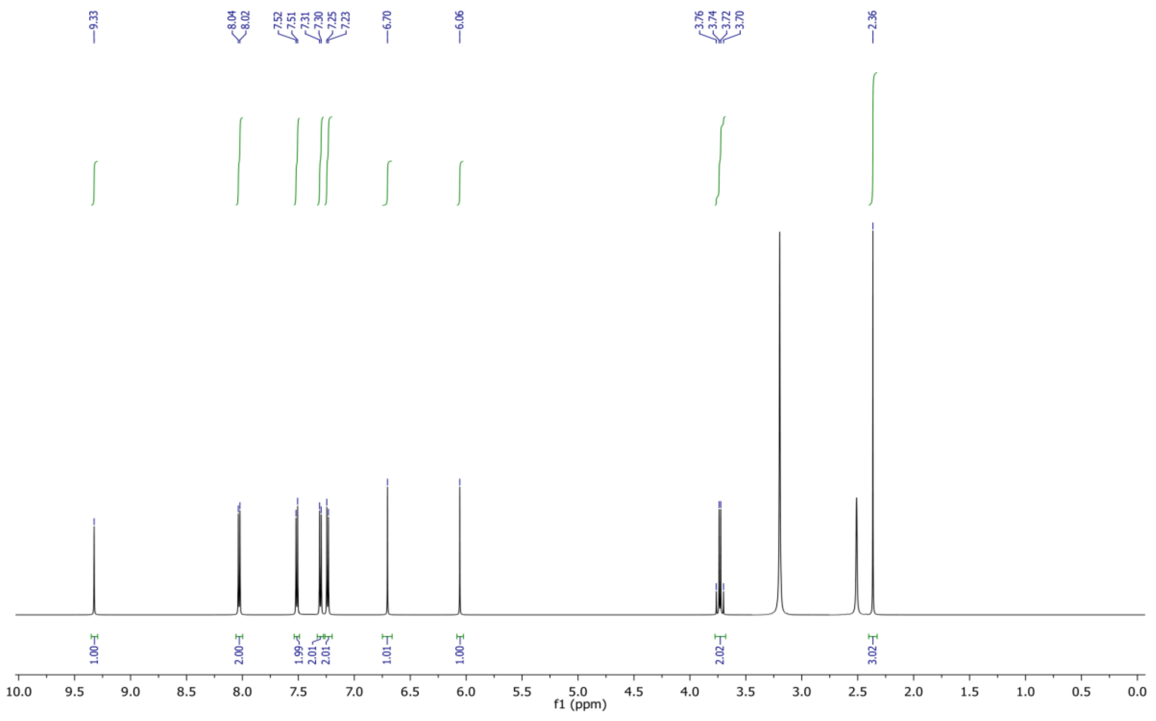

7i. ^{13}C NMR, 100 MHz, DMSO-d_6

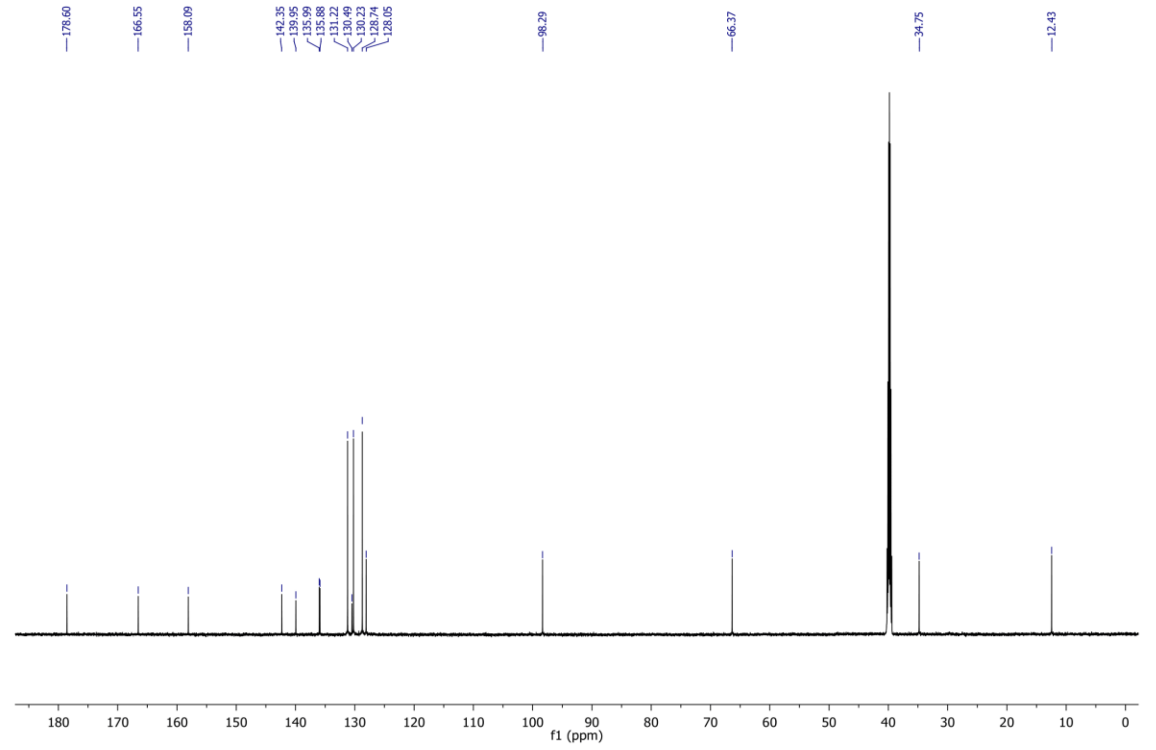

7i. HRMS

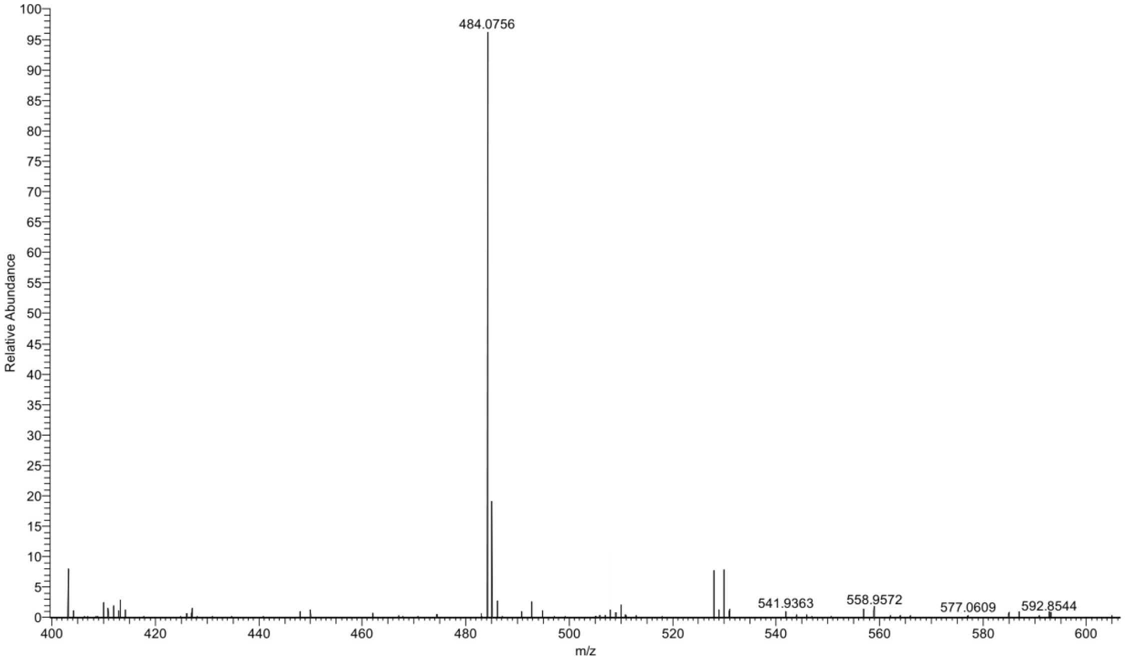

7j. ^{1}H NMR, 400 MHz, DMSO-d_6

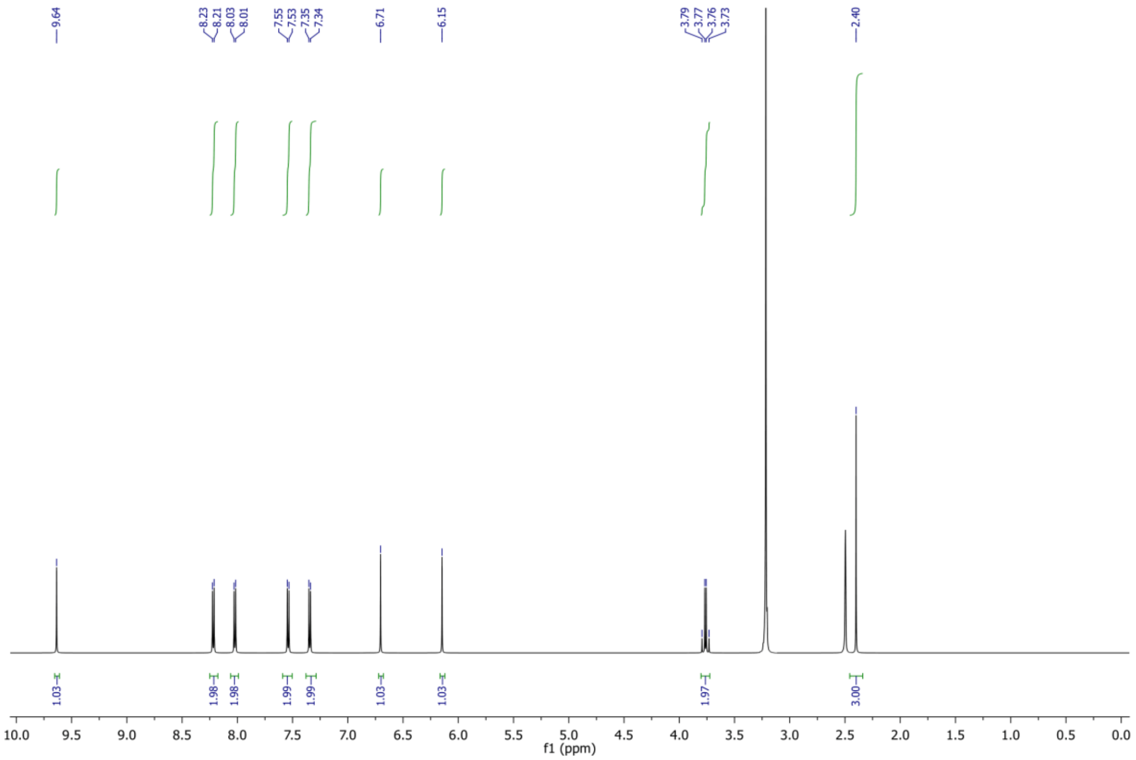

7j. ^{13}C NMR, 100 MHz, DMSO-d_6

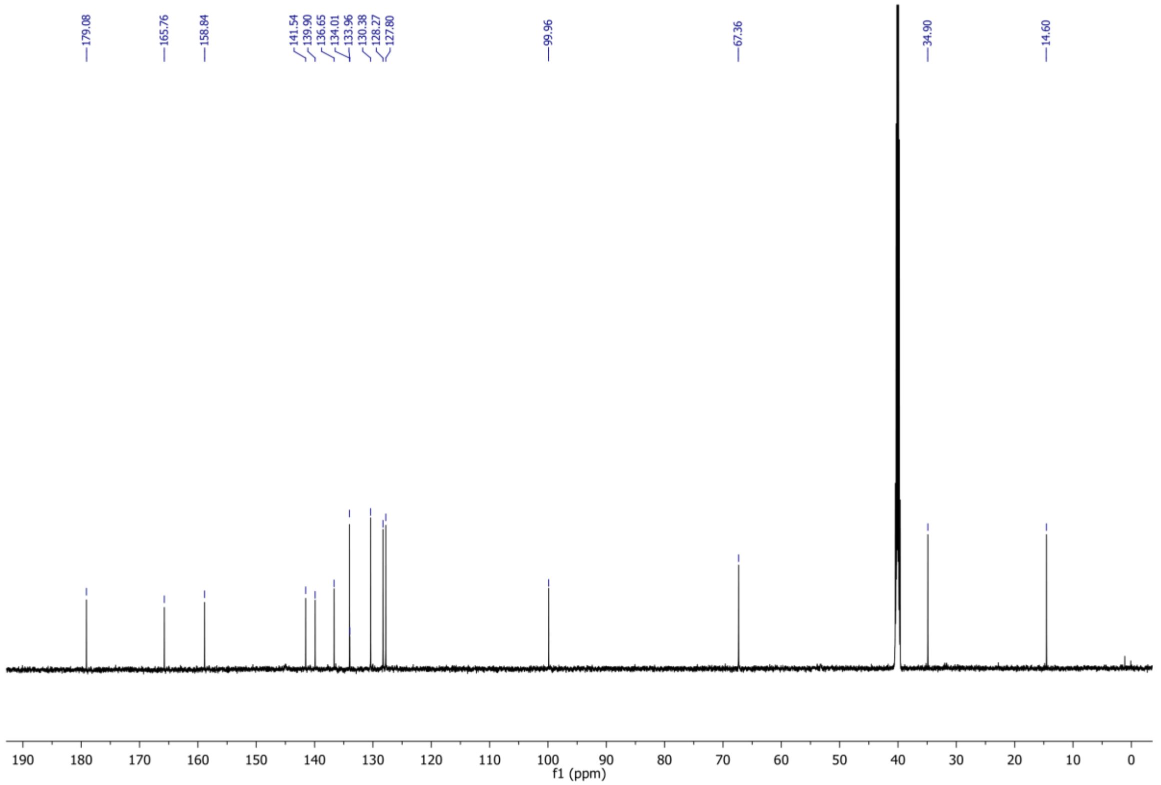

7j. HRMS

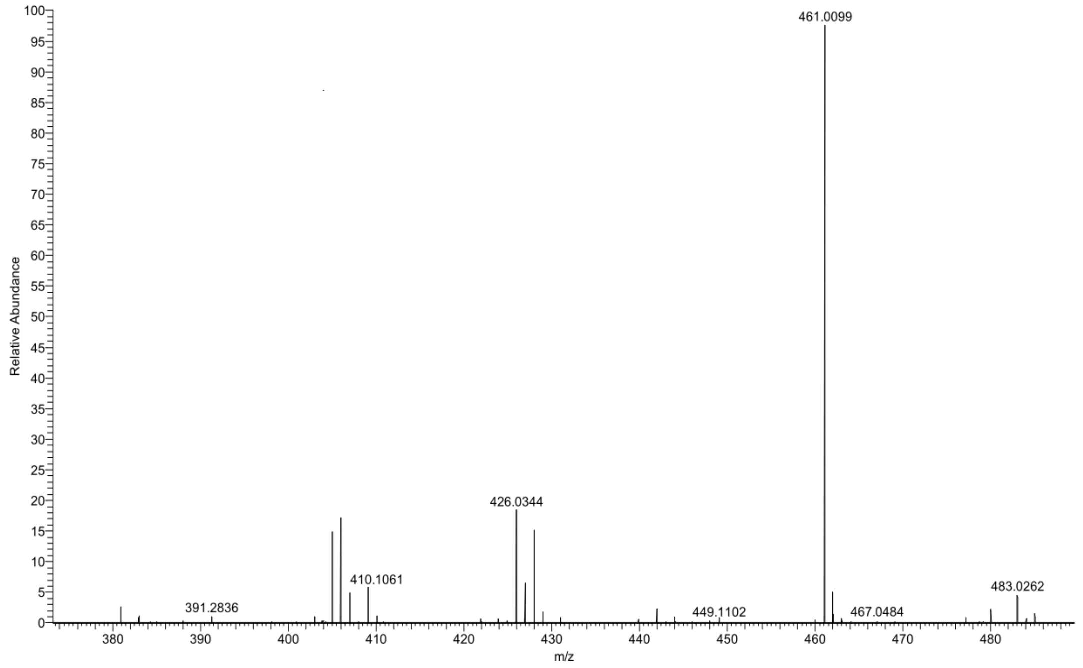

7k. ^{1}H NMR, 400 MHz, DMSO-d_6

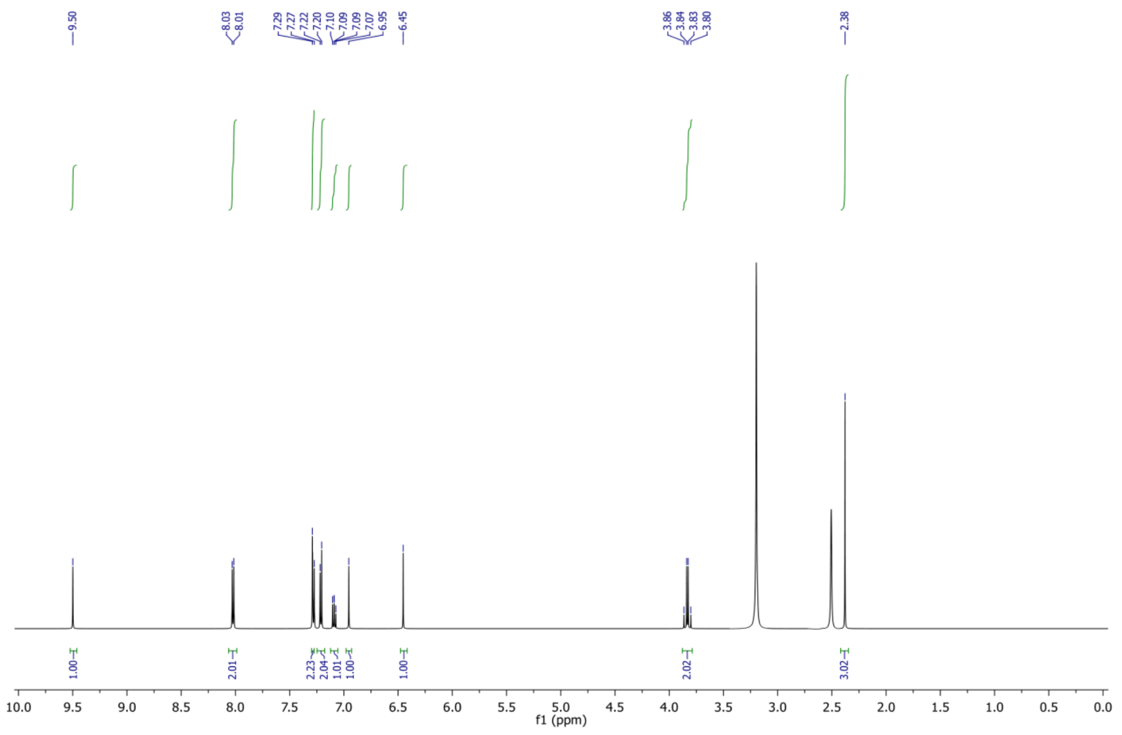

7k. ^{13}C NMR, 100 MHz, DMSO-d_6

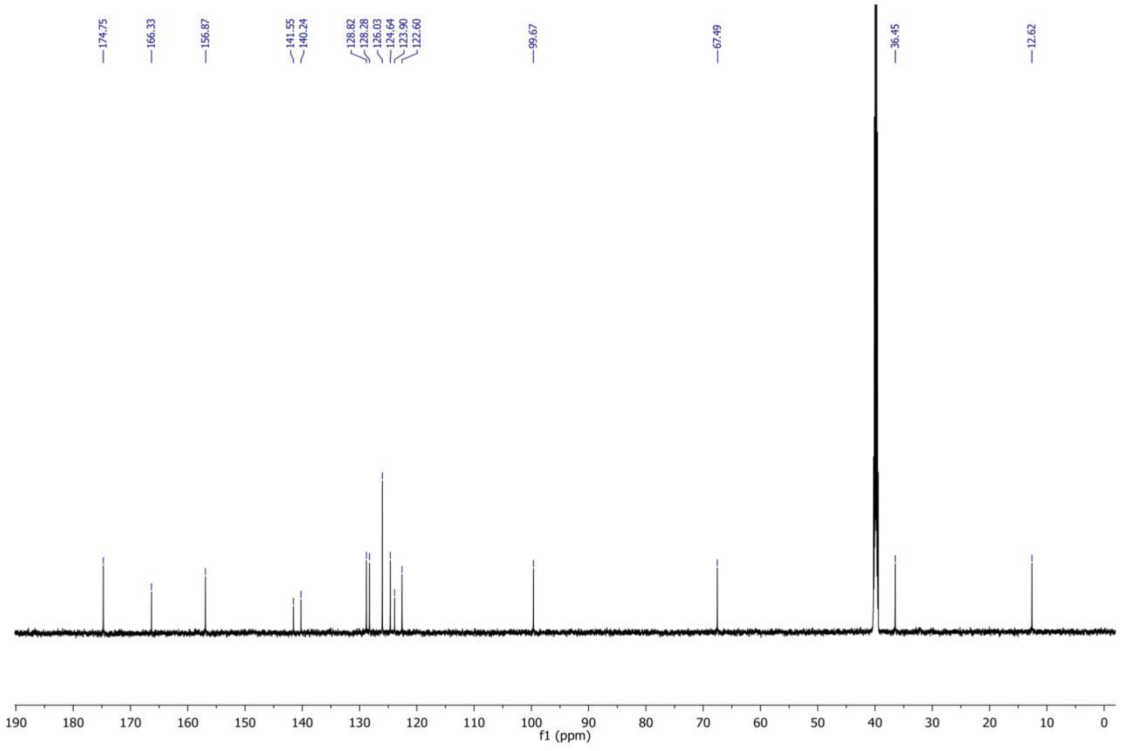

7k. HRMS

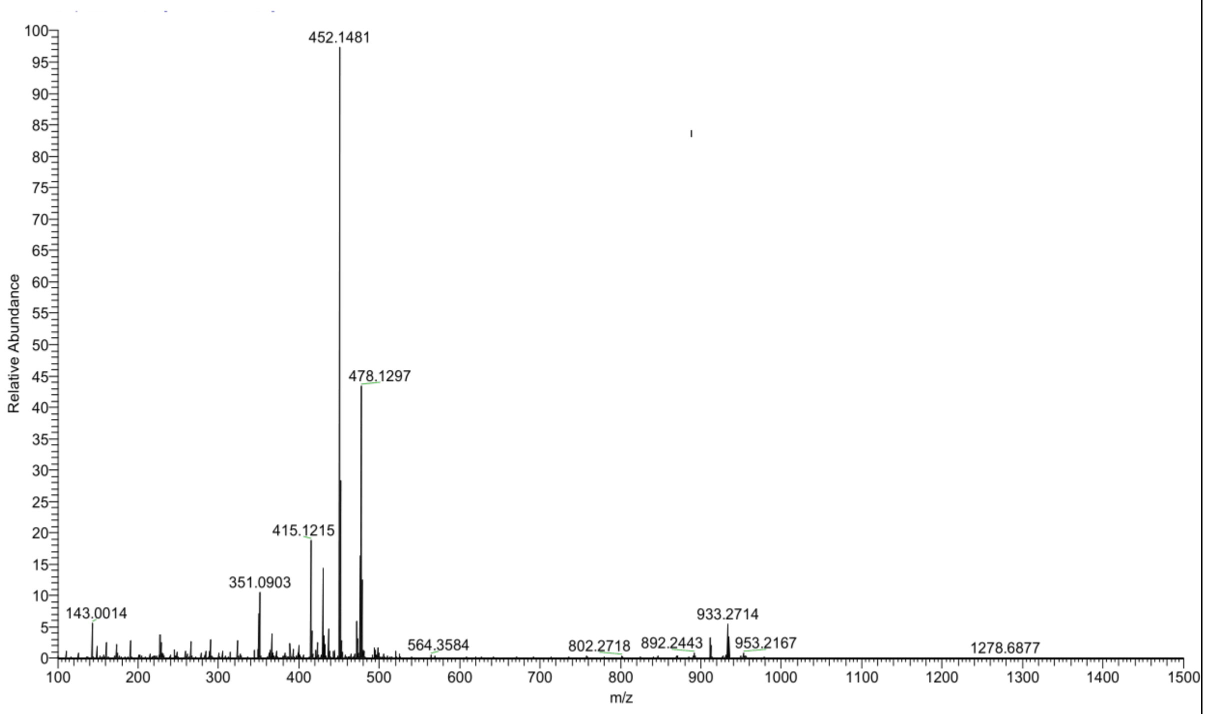

7l. ¹H NMR, 400 MHz, DMSO-d_6

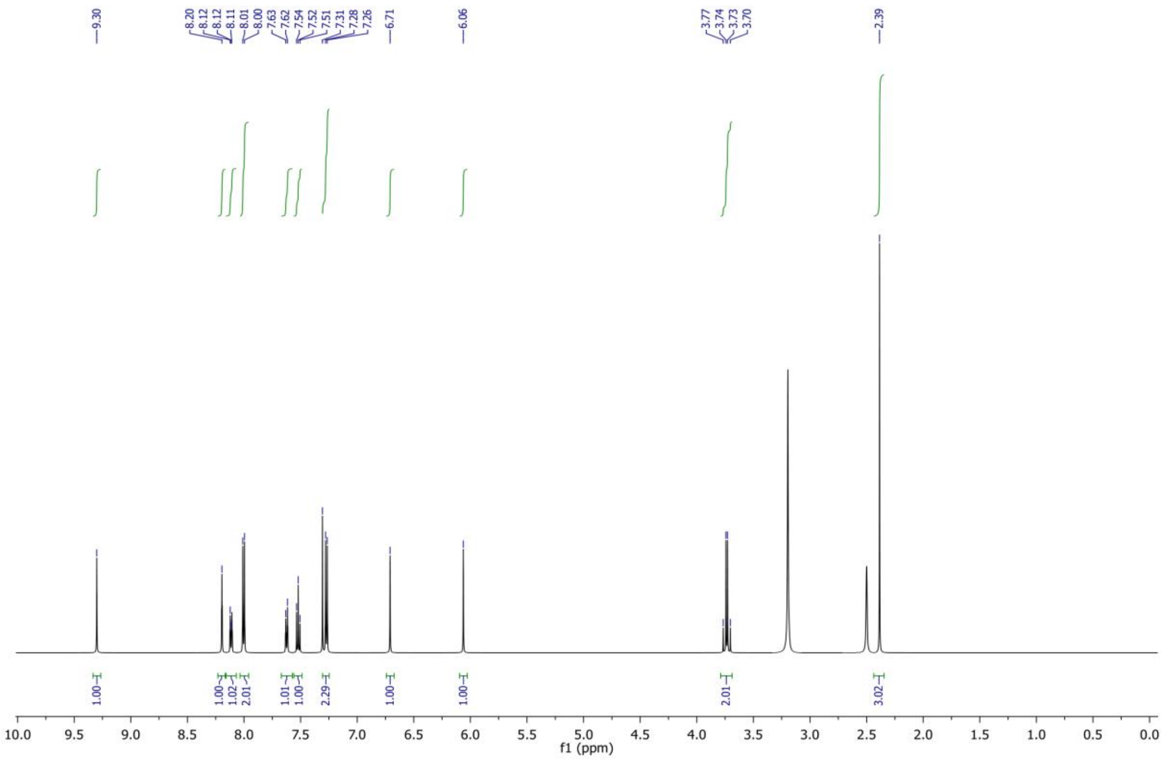

7l. ^{13}C NMR, 100 MHz, DMSO-d_6

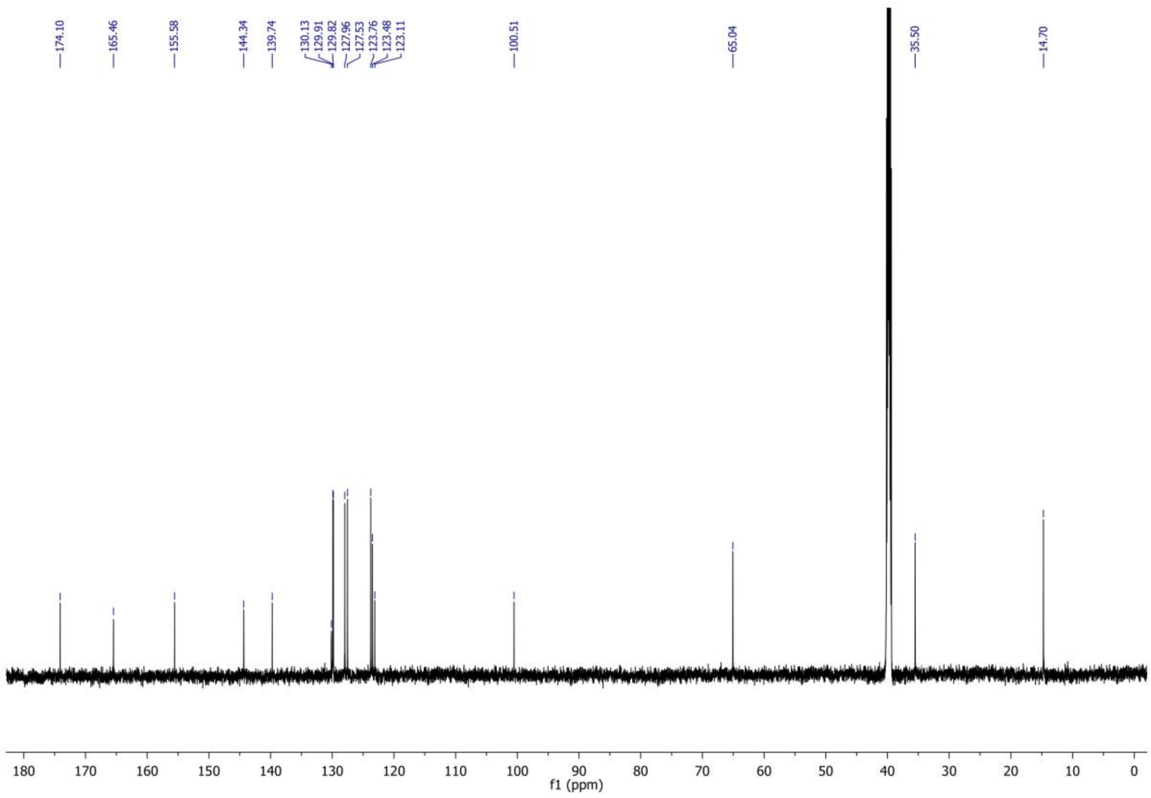

7l. HRMS

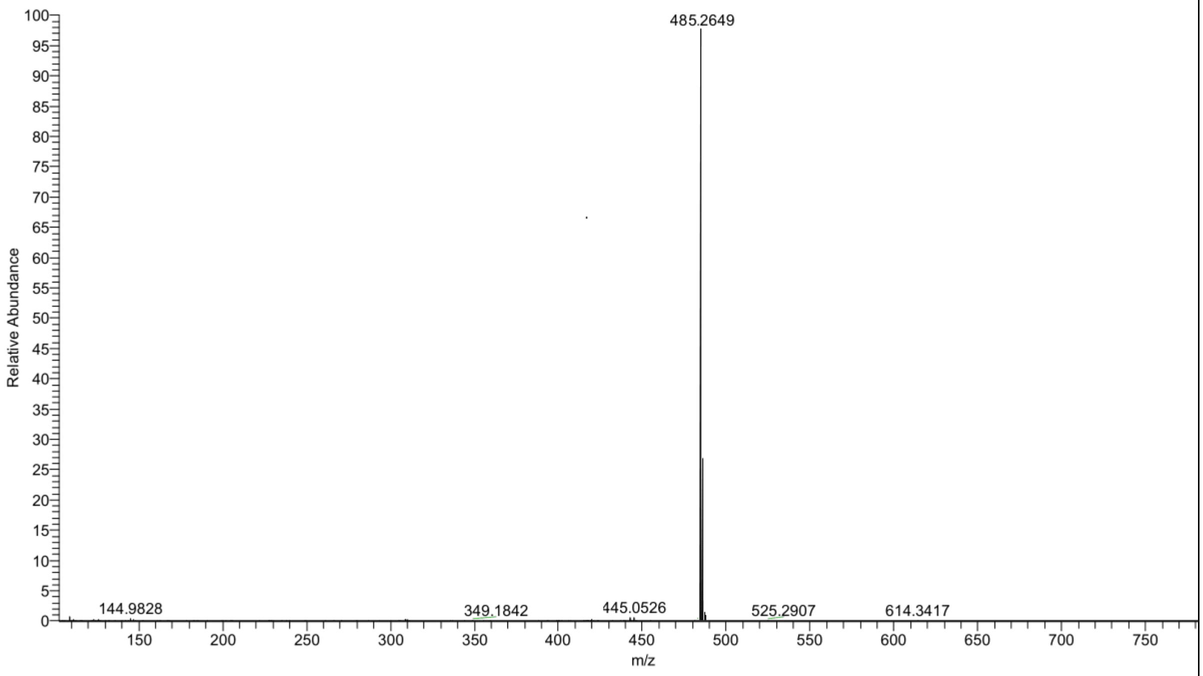

Supplement: Supplementary file 1 [file molecules-25-03570-s001.pdf]
